# Ambient solid-state mechano-chemical reactions between functionalized carbon nanotubes

**DOI:** 10.1038/ncomms8291

**Published:** 2015-06-15

**Authors:** Mohamad A. Kabbani, Chandra Sekhar Tiwary, Pedro A.S. Autreto, Gustavo Brunetto, Anirban Som, K.R. Krishnadas, Sehmus Ozden, Ken P. Hackenberg, Yongi Gong, Douglas S. Galvao, Robert Vajtai, Ahmad T. Kabbani, Thalappil Pradeep, Pulickel M. Ajayan

**Affiliations:** 1 Department of Materials Science and NanoEngineering, Rice University, Houston, Texas 77005, USA; 2 Department of Applied Physics, State University of Campinas, Campinas-SP 13083-959, Brazil; 3 DST Unit of Nanoscience and Thematic Unit of Excellence, Department of Chemistry, Indian Institute of Technology Madras, Chennai 600 036, India; 4 Department of Chemistry Rice University, Houston, Texas 77005, USA; 5 Department of Natural Science, Lebanese American University, P.O. Box 13-5053 Chouran, Beirut 1102 2801, Lebanon

## Abstract

Carbon nanotubes can be chemically modified by attaching various functionalities to their surfaces, although harsh chemical treatments can lead to their break-up into graphene nanostructures. On the other hand, direct coupling between functionalities bound on individual nanotubes could lead to, as yet unexplored, spontaneous chemical reactions. Here we report an ambient mechano-chemical reaction between two varieties of nanotubes, carrying predominantly carboxyl and hydroxyl functionalities, respectively, facilitated by simple mechanical grinding of the reactants. The purely solid-state reaction between the chemically differentiated nanotube species produces condensation products and unzipping of nanotubes due to local energy release, as confirmed by spectroscopic measurements, thermal analysis and molecular dynamic simulations.

## Abstract (web summary)

Direct coupling between chemical groups on individual nanostructures may lead to new architectures and reactions. Here, the authors report an ambient mechano-chemical reaction between two different reactant carbon nanotube varieties, which produces condensation products and unzipping of the nanotube structure.

## Graphical abstract

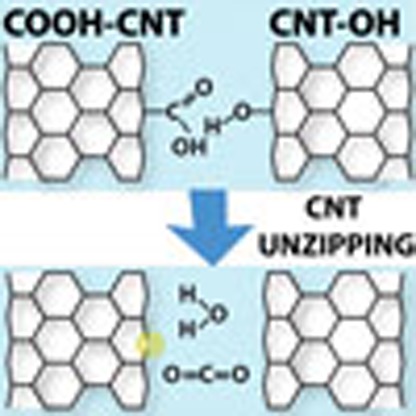

Chemical functionalization of nanoparticles can lead to their surface decoration with a variety of covalently attached functionalities to serve different goals such as drug delivery, cancer therapy, diagnostics and electronic devices^1^,^2^,^3^,^4^,^5^,^6^. Carbon nanotubes (CNTs) have been the subject of more than two decades of intense research^7^,^8^,^9^,^10^. Various approaches have been used to modify their surfaces via covalent and non-covalent attachments to change both physical and chemical properties. Activation of CNTs by the incorporation of COOH on the exterior surfaces by treating them with concentrated nitric acid has been widely used^11^,^12^. CNT-COOH can further be activated by acylation to form CNT-COCl, which can in turn be amidated or esterified^13^,^14^. CNTs carrying hydroxyl groups (CNT-OH) on their surfaces have also been synthesized by alkaline hydrothermal treatment of pristine nanotubes in alkaline medium^15^. Despite the remarkable CNT mechanical and electronic properties, their large use has been precluded by poor solubility in water or organic solvents, which favours bundle formation, thus limiting their chemical reactivity. Mechano-chemical reactions (MCRs) can be used to overcome such difficulties^16^,^17^. MCRs have been extensively used as synthetic protocols to obtain fullerene derivatives. Examples of these methods are fullerene dimers (C_120_), trimers (C_180_), cross dimers (C_60_-C_70_) and other fullerene derivatives obtained by the solid-state reactions with potassium salts such as KCN, K_2_CO_3_, reducing metals such as Mg and Al and solid aromatic amines under the high-speed vibration milling conditions^18^,^19^,^20^,^21^. MCRs of CNT functionalizations with other molecules such as C_60_, nitrenes, diazoinium compounds and metallic hydroxides under vigorous milling conditions have also been reported^22^,^23^,^24^,^25^. Another way of reacting fullerenes has been through their encapsulation into CNTs^26^. On the other hand, many methods have been used to produce graphene^27^,^28^,^29^,^30^,^31^, including the unzipping of nanotubes to make graphene nanoribbons. A typical chemical unzipping of CNTs makes use of oxidative techniques^31^ in concentrated acid (H_2_SO_4_) and post treatments with harsh reagents such as highly concentrated potassium permanganate (KMnO_4_). These processes involve harsh conditions to get to the final product (graphene), which can contain broken up or unzipped CNT. What has not been done is the use of nanotubes as solid-state reaction templates with specific chemical surface functionalities to induce direct coupling between the functional groups and concomitant breakdown of the cylindrical structure.

Here, we report the demonstration of unzipping of CNTs via a solid-state room temperature reaction between multiwalled CNTs (MWCNTs) containing different reactive functionalities of COOH and OH groups. The reaction is mechano-chemically induced, initiated at room temperature in ambient air, facilitated by the simple grinding of two chemically variant CNT reactants and leading to the unzipping of the nanotube (shown in Fig. 1a). By grinding equal weights of MWCNT-COOH and MWCNT-OH decorated with 1.41% and 0.46% by weight of COOH and OH (see experimental details of grinding and Supplementary Table 1), respectively^11^,^15^, a sheet-like lustrous material is formed spontaneously (Fig. 2a). Characterization of the material using different microscopic and spectroscopic methods, described later in the manuscript, suggests that the product consists predominantly of graphene or partially opened CNTs, possibly formed via the unzipping of the MWCNT reactants. The unzipping reaction may be represented by equation (1)


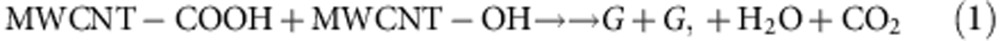


Where *G* and *G*′ represent the graphenes originating from the carboxylic and hydroxyl MWCNT (functionalized MWCNTs).

## Results

### Characterization

Attenuated total reflectance-Infrared Spectroscopy (ATR-IR) of the solid-state reaction product reveals almost complete absence of the COOH/O-H stretch in the region 3,600–2,800 cm^−1^ (Fig. 2a), in agreement with water formation during the reaction. Also, the intensity of the carbonyl band due to either carboxylic group or keto-enol tautomer in the CNT-OH diminishes significantly with the appearance of the adsorbed asymmetric CO_2_ mode at 2,345 cm^−1^. Compatible with these conclusions is the large decrease in the bending infrared mode of the CNTs at 868 cm^−1^ in the graphene product. The residual intensity is attributed to the unreacted CNTs. These results were further confirmed using X-ray photoelectron spectroscopic measurements.

In the C1s X-ray photoemission spectroscopy (XPS) of the MWCNTs, the signal at 289.2 eV corresponds to the carboxyl group, whereas the shoulders at 286.1 and 285.6 eV correspond to the C–O peak in MWCNT-COOH and MWCNT-OH, respectively^15^,^31^. Upon grinding, these features diminish in intensity and the most dominant peak becomes that of C=C at 284.8 eV, as seen in Fig. 2b. This is a strong evidence in favour of a condensation reaction taking place between the COOH and the OH. In addition, according to XPS, oxygen content drops from 0.715% in the unreacted mixture to 0.280% in the observed solid product (XPS data, Supplementary Table 1). The water formed comes from the OH of the carboxylic acid and constitutes half the oxygen of the carboxylic group. The fact that the loss of oxygen (0.715–0.280=0.435%) is appreciably larger than half the amount of oxygen of the carboxylic group or oxygen of the hydroxyl group in the unreactive mixture is in agreement with a graphene, H_2_O and CO_2_ reaction, the yield of which is ∼61% (Supplementary Table 1). This is comparable with the infrared data given before as well as with the simulation data presented later. To provide further evidence in favour of the graphene product from the solid-state reaction, we performed Raman spectroscopy of the reactant MWCNTs and that of the solid-state reaction product (Fig. 2c). All the bands in the product are upshifted relative to the reacting MWCNTs, whereas the second-order Raman band (2D) appearing at 2,705 cm^−1^ is downshifted as compared with the graphite band at 2,714 cm^−1^ (Fig. 2c). On the other hand, the 2D band in the product was well fitted by a sharp and symmetric Lorentzian in agreement with a few layer graphene-like product (Fig. 2c). The observations of lower 2D peak position relative to graphite, smaller *I*_D_/*I*_G_ ratio (0.201) and larger *I*_2D_/*I*_G_ ratio (1.21) for our reaction product are all in support of the formation of few layer graphene materials^32^,^33^.

Water formation during the solid-state reaction between the MWCNTs was also established through an *in-situ* mass spectrometric study of the reaction products. This was performed as described in Supplementary Note 1. Briefly, the experiment involved conducting the solid-state reaction in an enclosed mortar and pestle and sampling of the gases formed directly with a quadruple mass spectrometer, in the mass range of 1–300 amu. A blank measurement was done first without any CNTs in order to estimate the contribution from atmospheric gases and moisture. MWCNT-COOH and MWCNT-OH (1:1 ratio by weight) were then taken in the mortar and ground using a pestle. The gases in the reaction vessel were then allowed into the mass spectrometer inlet by opening a valve (Supplementary Fig. 1a). Intensity of the peak at *m*/*z* 18 (due to H_2_O^+^) increased significantly than in the blank experiment (Supplementary Fig. 1d). It is to be noted that there was no increase in intensity of nitrogen and oxygen ion currents. Increase in the H_2_O^+^ peak intensity with no increase in N^+^ intensity (derived from N_2_) clearly shows that this enhancement is due to the water resulting from solid-state condensation reaction between MWCNTs. No leak of atmospheric air would explain the increase in H_2_O^+^ as that would have resulted in an increase in N^+^ intensity as well. Corresponding mass spectral (intensity versus *m*/*z*) data are given in Supplementary Fig. 1b, which also show that only H_2_O^+^ intensity increased after the reaction while N^+^ and O^+^ intensities remained the same. To check whether this increase is due to the desorption of water vapour that was adsorbed on MWCNTs, a control experiment was carried out (Supplementary Fig. 1c). Initially, a blank was measured without MWCNTs. MWCNTs were then kept in the mortar without grinding for 2 min and gases inside were sampled (after the evacuation step). Intensities of N^+^, O^+^ and H_2_O^+^ were almost the same as that of the blank. After the mixture of MWCNTs was ground for 20 min, mass spectral measurement clearly showed an increase in intensity of only H_2_O^+^. The OH and COOH functionalized MWCNTs were separately ground and no increase in H_2_O^+^ was detected in those cases. This shows that H_2_O desorption from MWNTs is not the reason for increased H_2_O^+^ intensity. No increase in CO_2_ intensity was seen as it appears to be adsorbed effectively on the resulting graphene (see above).

Reproducibility of the results was confirmed by repeating these experiments several times with various ratios of the two MWCNT varieties. Furthermore, the decrease in the oxygen content, revealed from the XPS measurements, is supported by our mass spectrometric detection of water. Hence, the *in-situ* mass spectrometric experiments unambiguously give evidence for the solid-state condensation reaction between -COOH and -OH groups of the functionalized MWCNTs.

This was further supported with differential thermal analysis at different temperatures, which gave two distinct peaks, the more intense one occurs at ∼50 °C, whereas the less intense one occurs at ∼110 °C, shown in Supplementary Fig. 2. The peak at 110 °C is due to the desorption of water. As the energy evolved at lower temperature is appreciably higher than that due to desorption of water and in light of the strong CO_2_/graphene adsorption, the intense peak is assigned to the desorption of CO_2_. This conclusion is compatible with the infrared and XPS data presented before.

The proposed reaction from above spectroscopic and thermal measurements is further verified using imaging techniques such as scanning (SEM) and transmission electron microscopy (TEM). Figure 2e,f shows low and higher magnification SEM images revealing the structure of CNTs for the two functionalized raw materials. The amount of graphene-like material is negligible in these samples. On the other hand, the product powder shows predominantly sheet-like structure along with residues of partially reacted nanotubes (Fig. 2g,h). The image analyses of SEM images from different regions are used for calculating the amount of 2D sheets present and the residue of CNTs. The sheets are randomly distributed with a range of sizes with approximately 20% being unreacted CNT. To further confirm the opening of CNTs and the quality of the graphene-like product, TEM imaging was performed. Figure 3a shows bright-field image of the functionalized CNTs. It clearly shows CNTs to be multiwalled with an average diameter of 20 nm. The surfaces of the nanotubes appear disordered, due to heavy functionalization. Figure 3b shows large sheets of graphene-like material with smooth edges. The image shows multilayer structure. It is possible that the graphene flakes formed during the reaction have coalesced to form larger multilayer graphitic sheets. For further confirmation of the structure, selected area diffraction was performed. The result shows the hexagonal lattice of graphene stacks (shown as inset). Several images were taken for different samples of the product at different regions. Figure 3c,d shows products in the intermediate steps of the reaction. Several TEM images from different regions have been used for determining the number of layers in the multilayer sheets and the size of these graphene sheets. Histograms showing the size and number of layers formed due to the reaction are shown in Fig. 3e,f.

Kinetics of the reaction (and product formation) was monitored by measuring the intensity of the 2D Raman band of the graphene product at different temperatures^30^,^34^,^35^,^36^ (Supplementary Fig. 3). Arrhenius plot (detailed data given in Supplementary Table 2) of ln *k* versus 1/*T* (Supplementary Fig. 3) gives an activation energy of 16.63 kJ mol^−1^ (3.97 kcal mol^−1^), a value compatible with the activation energies reported for solid-state hydrogen bond-mediated proton-transfer reactions between many organic compounds such as carboxylic acid/phenol and carboxylic acid/amine combinations^37^,^38^,^39^,^40^,^41^,^42^,^43^,^44^.

### CNT unzipping reaction

**Figure 1:**
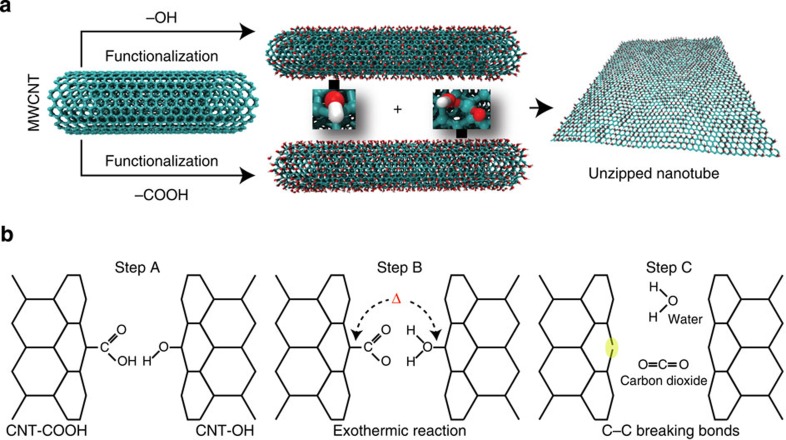
General scheme of the current work. (**a**) Solid-state synthetic unzipping scheme. (**b**) Hydrogen bond-mediated proton transfer unzipping mechanism: (Step A) hydrogen-bond formation and (Step B) fast proton-transfer, are followed by (Step C) water and CO_2_ as the products of the exothermic reaction. The released heat can induce the breaking of carbon-carbon bonds (highlighted yellow region) leading to unzipped tubes.

**Figure 2:**
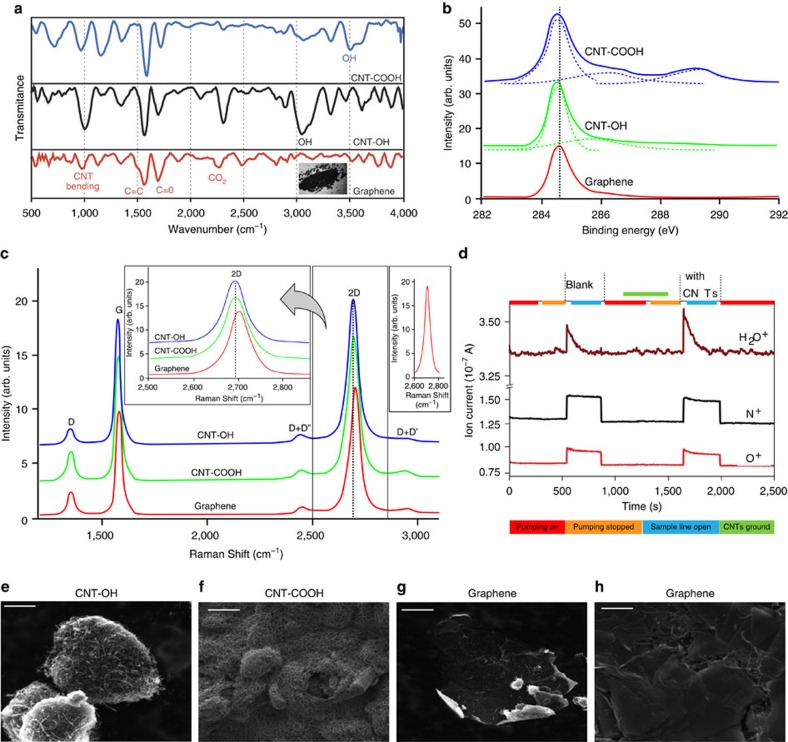
Materials characterization. (**a**) ATR-IR spectroscopy of the solid-state reaction graphene product after grinding (red) as compared with MWCNTs starting material MWCNT-COOH (blue) and MWCNT-OH (black). Formation of water in the unzipping process is confirmed by the absence of the COOH/OH stretch band in the 3,600–2,800 cm^−1^ region of the graphene product. The inset shows an image of the product of the solid-state reaction of MWCNT-COOH and MWCNT-OH. The image shows the thin lustrous sheets of the graphene product formed due to the unzipping of the MWCNTs. These sheets are covered and surrounded with some traces of the unreacted CNTs. (**b**) High-resolution C1s XPS spectrum of the solid-state reaction graphene product obtained after grinding (red) as compared with MWCNTs starting material (blue and green). (**c**) Raman spectroscopy of the solid-state reaction mixture after grinding (red) as compared with MWCNTs starting material (green and blue). Insets a 2D-band spectrum of the product as compared with those in the CNTs starting material, and a single-Lorentzian fit of the 2D band in the product. (**d**) Ion current versus time plots for N^+^, O^+^ and H_2_O^+^ obtained using *in-situ* mass spectrometric measurements during the solid-state condensation reaction between MWCNTs. SEM image of the two reactants (**e**) CNT-COOH, scale bar, 2 μm; and (**f**) CNT-OH, scale bar, 2 μm. (**g**,**h**) The graphene nanosheet product along with residue of CNTs at two magnifications, scale bar: (**g**) 5 μm, (**h**) 1 μm.

**Figure 3:**
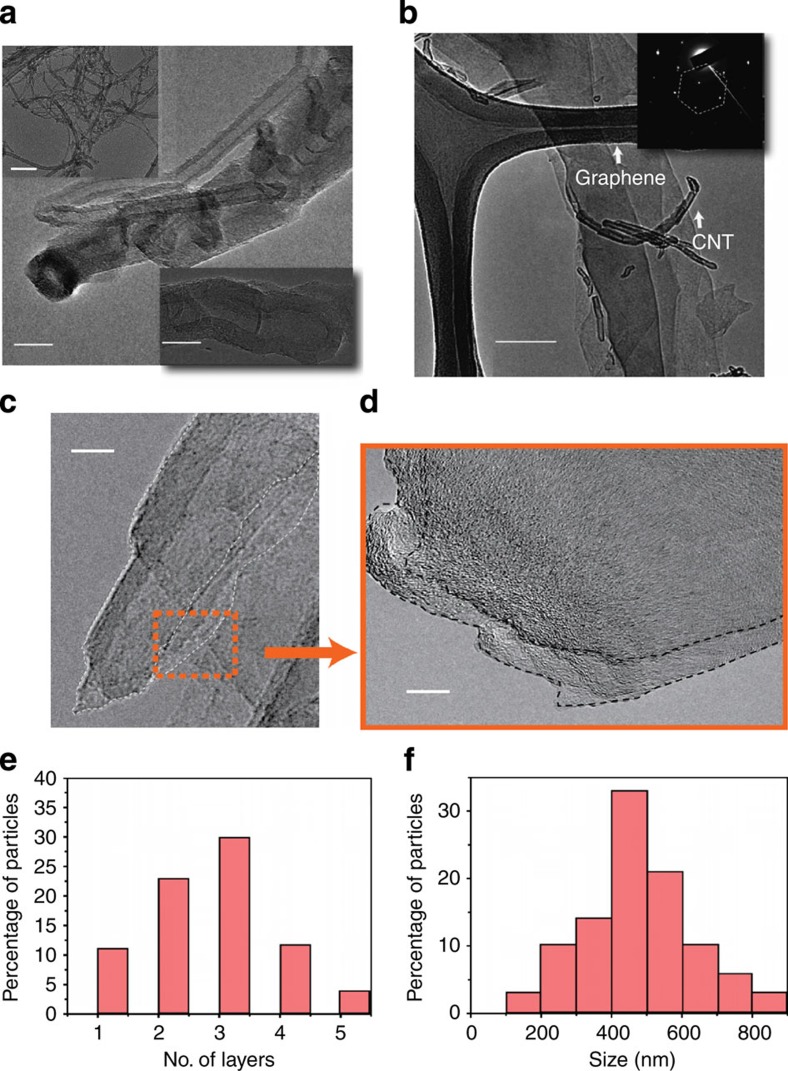
TEM characterization. Bright-field TEM micrograph of the reactant (**a**) CNTs, scale bar, 20 nm, with insets showing low-magnification image, scale bar, 200 nm and another HRTEM image, scale bar, 5 nm. HRTEM, high-resolution transmission electron microscopy. (**b**) Graphene prepared using the current method, scale bar, 200 nm; inset showing diffraction pattern. The image shows multilayer structure. The selected area diffraction performed shows the hexagonal symmetry of graphene. The high-resolution image of these layered structures shows the hexagonal lattice arrangement of graphene. (**c**,**d**) The images of partially unzipped CNTs with different magnifications. Scale bar: (**c**) 10 nm, (**d**) 5 nm. (**e**,**f**) Histogram of number of layers and size, respectively, of the graphene sheets produced using the current method.

In light of the above, *we* suggest that the CNT unzipping reaction consists of a slow step that brings the CNTs together through mechanical grinding allowing the COOH and OH groups to react (Step A in Fig. 1b). Accordingly, this step is followed by fast proton transfer (Step B in Fig. 1c) from the carboxylic group to the hydroxyl group to form [MWCNT-OH_2_]^+^and [MWCNT-COO]^−^, whose exothermic reactions produce water and CO_2_ (Step C in Fig. 1d). The energy released can induce carbon-carbon bond breaking (highlighted yellow region) leading to unzipped structures.

**Figure 4:**
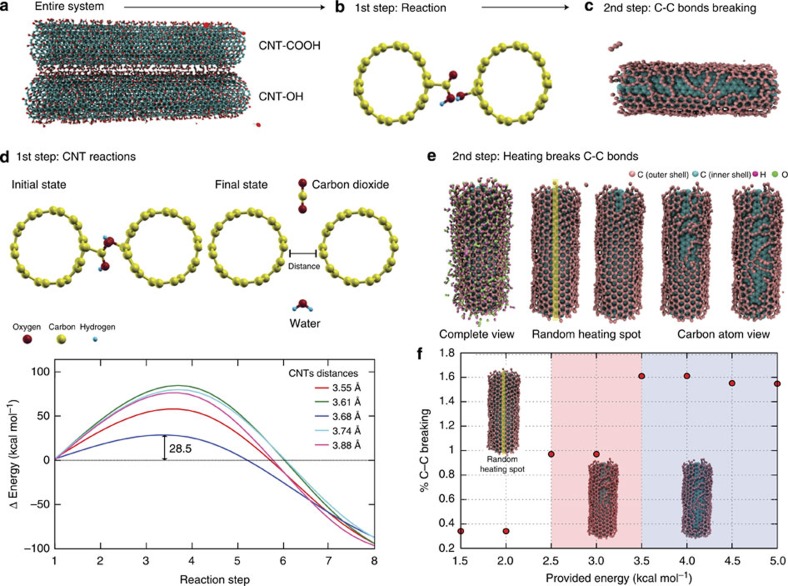
Simulation of the process. (**a**) General scheme for the simulated unzipping process; (**b**) functionalized tubes (CNT-COOH and CNT-OH); (**c**) initial configuration for energy barrier reaction calculation and resultant unzipped nanotubes. (**d**) Reactants (different functionalized tubes) and products (tubes, water and carbon dioxide) of the simulated reaction and the corresponding energy barrier values for different tube separations. (**e**) Schematic view of simulated unzipping process: Initial configuration showing the functionalized tube. For better visualization, the CNT functional groups were made transparent in the following four representative unzipping steps. The yellow line indicates the region where the heating spot was applied. (**f**) The percentage of C–C bond breaking and the corresponding final configuration for different energies of the heating spot.

Owing to the complexity of this process, simulations are divided into two parts. The first one is related to the estimation of the energy barriers associated with the chemical reactions between the CNT functional groups (value and determination of the thermodynamical character, exothermic or endothermic). In the second part, we have estimated the minimum required energy (threshold values, using ReaxFF^45^,^46^) value to trigger the unzipping (breaking carbon bonds). The unzipping can produce partially or totally opened tubes, thus generating the nanoribbons. The obtained value was then contrasted against the estimated energy released during the chemical reactions. This approach is schematically presented on Fig. 4a–c. The barrier results (Fig. 4d) were obtained through nudged elastic band (NEB) simulations. NEB is one of the standard methods used to determine energy barrier height and the chemical pathways of chemical reactions, assuming the reactants and products are known^47^,^48^,^49^. Density functional theory calculations (see Supplementary Methods) support the experimental interpretation that water and carbon dioxide are the final products of the reactions between the CNT functional groups (initial and the final states are presented in Fig. 4d).

Energy barrier calculations were performed for different CNT distances (3.5 to 4.0 Å, with stepsize increments of 0.05 Å; see Supplementary Fig. 5). These values were chosen over a range where it is expected that reaction (3.5 Å) and no reaction (4.0 Å) can occur. Some of these curves are shown in Fig. 4d. The slight differences from the reference values (for instance, 3.88 instead of 3.90) are a consequence of the initial geometry optimization process. Our results indicate that the lowest energy barrier can occur for tubes separated by ∼3.7 Å, with an associated energy barrier height around 30 kcal mol^−1^. More detailed information can be obtained from the Supplementary Movie 1.

From these results, we can conclude that these reactions need to be energy assisted. The energy needed to overcome the reaction barriers of CNTs opening can be provided by the exothermic reaction between the carboxylic acid and alcohol functionalities, which in turn provides necessary amount of energy for the cleavage of carbon–carbon bonds and consequently the unzipping of the CNTs. The main mechanical grinding effect is only to bring the CNTs closer. Maximizing the contact area through total or partial axial tube alignment during the grinding, appears to be the key to maximize the number of reactions to produce totally or partially unzipped tubes, which is consistent with the available experimental data. Our results also showed that this reaction is exothermic, which is in agreement with more refined density functional theory calculations that indicate that the released energy is about 25 kcal mol^−1^ (see Supplementary Methods for details). This injected energy can result in thermal (increasing the temperature around the region where the reaction occurred) and/or mechanical (eventual C–C bond breaking) effects, thus leading to tube unzipping, as recently demonstrated by molecular dynamics (MD) simulations^50^.

In the second step, to estimate the minimum amount of energy needed to trigger the unzipping process, we have carried out a systematic MD study. Our models were composed of MWCNTs, where for simplicity of the inner-layers are kept frozen. This approach has been proved effective in the unzipping of carbon^51^ and boron nitride^52^ tube studies. To mimic the injected energy generated by the chemical reactions, we used the so-called heating spot protocol^53^,^54^ (LAMMPS Software manual ( http://lammps.sandia.gov)). Using this protocol, we randomly added non-translational kinetic energy (heat) to selected atoms within a subregion defined as ‘contact area' (highlighted strip in Fig. 4e). The atoms belonging to the ‘contact region' can be thought as the ones that would be in contact with an adjacent tube during the grinding procedure. The investigated injected energy range was from 1.0 up to 30 kcal mol^−1^ (the barrier values). These results are presented in Fig. 4f (limited to the range of 1.5 up to 5.0 kcal mol^−1^). The percentage of C–C broken bonds is shown for each heating spot value. When the heat transferred to the system is up to 2.0 kcal mol^−1^, the amount of broken bonds is around 0.3%, which is not enough to produce the tube unzipping. Increasing the heat up to 3.0 kcal mol^−1^, the number increases to 1.0%, which is enough to create some defects in the tube (red shaded region—Fig. 4f). When the heat delivered to the system is higher than 3.5 kcal mol^−1^, the amount of broken bonds reaches 1.6%, which is enough to break up the tube along its main axial direction and to produce the unzipping effect (grey shaded region– Fig. 4f). These results show that only a small fraction (in this case, only 14%) of the estimated energy released by the chemical reactions would be enough to provide C–C broken bonds and to trigger the unzipping process.

## Discussion

In conclusion, we have reported, for the first time, ambient solid-state mechano-chemical (through simple mechanical grinding) reactions between CNT-COOH and CNT-OH resulting in unzipped nanotubes. The released heat during the process results in C–C bond breaking, which subsequently leads to CNT unzipping. The proposed method can be used as a generic approach to develop new theoretical and synthetic frameworks where reactions could be designed and controlled via chemically modified solid reactants.

## Methods

### Synthesis and reactions of CNTs

MWCNTs) were prepared by using water-assisted chemical vapour deposition (WACVD) on a Si substrate. Si wafer, on which 10 nm aluminum and 1.5 nm iron layers were deposited via electron beam, was placed in a quartz tube and heated to 775 °C in Ar/H_2_ buffer having 15% of H_2_-ethylene gas and water vapour were introduced in the quartz tube for 30 min.

Iron impurity from the nanotubes was removed by suspending them overnight in 2.6 M nitric acid at 120 °C. After that the nanotubes were separated by filtration using 0.2 μm GTTP Millipore membrane and washed extensively with water and dried. MWCNT-COOH and MWCNT-OH were prepared from pristine CNTs according to the procedures in refs 1, 3, respectively.

The unzipping reaction was done by grinding equal weights of MWCNT-COOH and MWCNT-OH. In current experiment, two different sizes of mortars and pestles (made up of Traditional Agate) with radii of 2.5 and 5 cm were used. Initial experiments are performed using mortar of 5 cm radii and then the same has been repeated for 2.5 cm radii. The samples were collected at different time duration (5, 10, 15 and 20 min). Among all these, the 20-min grinding shows best yield, which is reported in the manuscript. We start observing change in the initial time itself but as the duration of grinding increases, the yield increases as can be observed as lustrous sheets are formed during grinding. As for the reproducibility of the results, the reaction was reproduced in three different labs in three different countries (Professor Ajayan's Lab in Houston (USA), Professor Pradeep's lab in India (Chennai) and Professor Ahmad Kabbani's lab in Lebanon (Beirut)) by different colleagues and collaborators involved in this paper. As for the environment and temperature, this reaction was done in different environments under vacuum, different labs (that has controlled humidity and temperature) and also outside in open air as well in variable humidity, different temperatures and in different times, seasons and countries (Houston (USA), India (Chennai), Lebanon (Beirut)).

### Materials characterization

Raman spectra were measured using a Renishaw Raman spectrometer at 514.5 nm excitation. The variable temperature Raman study was carried out by bringing MWCNT-COOH and MWCNT-OH to same temperatures before grinding them. Grinding was done in a separate vessel at the same temperature. All reaction mixtures were quenched to room temperature before Raman spectra were recorded. 2D line intensities at different temperatures were normalized to the same 2G line intensities. XPS spectra were measured using a Surface Science Instrument SSX-100. SEM image was obtained by using FEI Quanta 400 SEM. TEM image was obtained using a JEOL 2100F TEM. Imaging has been performed using field emission electron microscope with a low current density and low accelerating voltage. Mass spectrometric measurements were carried out using a residual gas analyser (BalzersThermostar with Quadstar 32 bit software). This instrument is basically a residual gas analyser, equipped with an electron impact ionization source and a quadruple mass analyser. For measurements, we utilized multiple ion detection modes. In multiple ion detection, we measured ion current as a function of time.

### Simulation methodology

We used the ReaxFF force field with parameter set from refs 45, 46. This parameter set is optimized for structures containing the elements carbon, hydrogen and oxygen, thus appropriated to our systems. For the NEB calculations, we considered eight replicas between the initial and final configurations. The ‘spring' that connects each replica has a constant force of 10 kcal /mol^−1^ Å^−1^. The adopted criterion for NEB convergence was energy differences below 1.0 × 10^−4^ kcal mol^−1^. All the calculations using ReaxFF and/or NEB were carried out using the LAMMPS code^52^,^53^. For the MD simulations of the CNT unzipping processes, the addition of heat (through the heat spot protocol) into the system is performed every 1.0 ps during 50 ps. The heat is added to the atoms belonging to a circular (4 Å radius value). We also tested different circle radii values, ranging from 3 to 6 Å, but no significant differences in the results were observed.

## Additional information

**How to cite this article:** Kabbani, M. A. *et al.* Ambient solid-state mechano-chemical reactions between functionalized carbon nanotubes. *Nat. Commun.* 6:7291 doi: 10.1038/ncomms8291 (2015).

## Supplementary Material

Supplementary Figures, Supplementary Tables, Supplementary Note, Supplementary Methods and Supplementary References. Supplementary Figures 1-5, Supplementary Tables 1-2, Supplementary Note 1, Supplementary Methods and Supplementary References

Supplementary Movie 1 Simulated Reaction between the CNT functional groups: Simulated reaction between the CNT functional groups (-COOH and –OH) resulting in water and carbon dioxide. The tubes are placed at the optimized distance (d=3.68Å) that yields the lowest energy barrier.

## References

[b1] Schulz-Dobrick M., Sarathy K. V. & Jansen M. Surfactant-free synthesis and functionalization of gold nanoparticles. J. Am. Chem. Soc. 127, 12816–12817 (2005). 16159272 10.1021/ja054734t

[b2] Zatas M., Katz E., Baron R. & Willner I. Reconstitution of Apo-Glucose dehydrogenase on pyrroloquinoline quinine-functionalized Au nanoparticles yields an electrically contacted biocatalyst. J. Am. Chem. Soc. 127, 12400–12406 (2005). 16131222 10.1021/ja052841h

[b3] Wang L., Wang L., Zhu C., Wei X. & Kan X. Preparation and application of functionalized nanoparticles of CdS as a fluorescence probe. Anal. Chim. Acta. 468, 35–41 (2005).

[b4] Shenhar R., Norsten T. B. & Rotello V. M. Polymer-mediated nanoparticle assembly: Structural control and applications. Adv. Mater. 17, 657–669 (2005).

[b5] Liu H. & Alivisatos A. P. Preparation of asymmetric nanostructures through site selective modification of tetrapods. Nano Lett. 4, 2397–2401 (2004).

[b6] Tomalia D. A. Birth of a new macromolecular architecture: dendrimers as quantized building blocks for nanoscale synthetic polymer chemistry. Prog. Polym. Sci. 30, 294–324 (2005).

[b7] Iijima S. Helical microtubules of graphitic carbon. Nature 354, 56–58 (1991).

[b8] Jariwala D., Sangwan V. K., Lauhon L. J., Marks T. J. & Hersam M. C. Carbon nanomaterials for electronics, optoelectronics, photovoltaics, and sensing. Chem. Soc. Rev. 42, 2824–2860 (2013). 23124307 10.1039/c2cs35335k

[b9] Jeng E. S., Moll A. E., Roy A. C., Gastala J. B. & Strano M. S. Detection of DNA hybridization using the near-infrared band-gap fluorescence of single-walled carbon nanotubes. Nano Lett. 6, 371–375 (2006). 16522025 10.1021/nl051829k PMC6438164

[b10] Kovtyukhova N. I., Mallouk T. E., Pan L. & Dickey E. C. Individual single-walled nanotubes and hydrogels made by oxidative exfoliation of carbon nanotubes ropes. J. Am. Chem. Soc. 125, 9761–9769 (2003). 12904042 10.1021/ja0344516

[b11] Liu J. *et al.* Fullerene pipes. Science 280, 1253–1256 (1998). 9596576 10.1126/science.280.5367.1253

[b12] Worsley K., Kondrat R., Pal S., Kalinina I. & Haddon R. Isolation and identification of low molecular weight carboxylated carbons derived from the nitric acid treatment of single-walled carbon nanotubes. Carbon 49, 4982–4986 (2011).

[b13] Niyogi S. *et al.* Chemistry of single-walled carbon nanotubes. Acc. Chem. Res. 35, 1105–1113 (2002). 12484799 10.1021/ar010155r

[b14] Liu Z. *et al.* Organizing single-walled carbon nanotubes on gold using chemical self-assembling techniques. Langmuir 16, 3569–3573 (2000).

[b15] Yang D., Guo G., Hu J., Wang C. & Jiang D. Hydrothermal treatment to prepare hydroxyl group modified multi-walled carbon nanotubes. J. Mater. Chem. 18, 350–354 (2008).

[b16] Zhu S. E., Fei F. L. & Wang G. W. Mechanochemistry of fullerene and related materials. Chem. Soc. Rev. 42, 7535–7570 (2013). 23677148 10.1039/c3cs35494f

[b17] Drexler K. E. Nanosystems: Molecular Machinery, Manufacturing, and Computation John Wiley and Sons (1992).

[b18] Wang G. W., Komatsu K., Murata Y. & Shiro M. Synthesis and X-ray structure of dumb-bell-shaped C_120_. Nature 387, 583–584 (1997).

[b19] Komatsu K., Fujiwara K., Tanaka T. & Murata Y. The fullerene dimer C_120_ and related carbon allotropes. Carbon 38, 1529–1532 (2000).

[b20] Kunitake M. *et al.* First structural analysis of C_60_ trimers by direct observation with STM. Angew. Chem. Int. Ed. 41, 969–972 (2002). 10.1002/1521-3773(20020315)41:6<969::aid-anie969>3.0.co;2-i 12491284

[b21] Komatsu K., Fujiwara K. & Murata Y. The fullerene cross dimer C130: Synthesis and properties. Chem.Commun. 2000, 1583–1584 (2000).

[b22] Li X. *et al.* C60 modified single-walled carbon nanotubes. Chem. Phys. Lett. 377, 32–36 (2003).

[b23] Holizinger M. *et al.* Functionalization of single-walled carbon nanotubes with (R-) oxycarbonylnitrenes. J. Am.Chem.Soc. 125, 8566–8568 (2003). 12848565 10.1021/ja029931w

[b24] Dyke C. A. & Tour J. Solvent-free functionalization of carbon nanotubes. J.Am.Chem.Soc. 125, 1156–1158 (2003). 12553803 10.1021/ja0289806

[b25] Pan H. *et al.* Carbon nanotubes from mechanochemical reaction. Nano Lett. 3, 29–32 (2003).

[b26] Smith B. M., Monthiooux M. & Luzzi D. L. Encapsulated C60 in carbon nanotubes. Nature 396, 323–325 (1998).

[b27] Novoselov K. S. *et al.* Electric field effect in atomically thin carbon films. Science 306, 666–669 (2004). 15499015 10.1126/science.1102896

[b28] Coleman J. N. *et al.* Two-dimensional nanosheets produced by liquid exfoliation of layered materials. Science 331, 568–571 (2011). 21292974 10.1126/science.1194975

[b29] Hersam M. C. *et al.* Chemically resolved interface structure of epitaxial graphene on SiC (0001). Phys. Rev. Lett. 111, 215501 (2013). 24313501 10.1103/PhysRevLett.111.215501

[b30] Jiao L., Wang X., Diankov G. & Dai H. Narrow graphene nanoribbons from carbon nanotubes. Nature 458, 877–880 (2009). 19370031 10.1038/nature07919

[b31] Kosynkin D. V. *et al.* Longitudinal unzipping of carbon nanotubes to form graphene nanoribbons. Nature 458, 872–876 (2009). 19370030 10.1038/nature07872

[b32] Graf D. *et al.* Spatially resolved Raman spectroscopy of single and few layer graphene. Nano Lett 7, 238–242 (2007). 17297984 10.1021/nl061702a

[b33] Ferrari A. C. & Basko D. M. Raman spectroscopy as a versatile tool for studying the properties of graphene. Nat. Nanotechnol. 8, 235–246 (2013). 23552117 10.1038/nnano.2013.46

[b34] He R. *et al.* Observation of low energy raman modes in twisted bilayer graphene. NanoLett 13, 3594–3601 (2013). 10.1021/nl4013387 23859121

[b35] Ruoff R. *et al.* Selective-area fluorination of graphene with fluoropolymer and laser irradiation. Nano Lett. 12, 2374–2378 (2012). 22482878 10.1021/nl300346j

[b36] Ferrari A. C. *et al.* Raman spectrum of graphene and graphene layers. Phys. Rev. lett 97, 187401 (2006). 17155573 10.1103/PhysRevLett.97.187401

[b37] Stevens J. *et al.* Proton transfer and hydrogen bonding in the organic solid state. Phys. Chem 16, 1150–1160 (2014). 10.1039/c3cp53907e 24292812

[b38] Koeppe B., Tolsey P. & Limbach H. Reaction pathways of proton transfer in hydrogen-bonded phenol-carboxylate complexes. J. Am. Chem Soc. 133, 7897–7908 (2011). 21534587 10.1021/ja201113a

[b39] Hynes J., Klinman J., Limbach H. & Schowen R. Hydrogen Transfer Reactions 1–4WILEY-vch (2007).

[b40] Bertran J. F., Alvarez J. & Reguera E. Proton transfer in the solid state: Reactions of organic acids and amines. Solid State Ionics 106, 129–135 (1998).

[b41] Fernández-Bertrán J. & Reguera E. Proton transfer in the solid state: mechanochemical reactions of fluorides with acidic substances. Solid State Ionics 112, 351–354 (1998).

[b42] Vinogradov S. N. & Linnel R. H. Hydrogen bonding Von Nostrand Reinhold Company (1971).

[b43] Steiner T. & Saenger W. Lengthening of the covalent O--H bond in O--H...O hydrogen bonds. reexamination from low-temperature neutron diffraction data of organic compounds. ActaCrystallogr B50, 348–357 (1994).

[b44] Limbach H. H., Deniisov G. S. & Golubev N. S. Hydrogen Bond Isotope Effects Studied by NMR, in isotopes effects in the biological and chemical sciences eds Kohen A., Limbach H. H. Ch 7, Taylor and Francis (2005).

[b45] VanDuin A. C. T., Dasgupta S., Lorant F. & Goddard W. A. ReaxFF: a reactive force field for hydrocarbons. J. Phys. Chem A 105, 9396–9409 (2001).

[b46] Chenoweth K., van Duin A. C. T. & Goddard W. A. ReaxFF reactive force field for molecular dynamics simulations of hydrocarbon oxidation. J. Phys. Chem. A 112, 1040–1053 (2008). 18197648 10.1021/jp709896w

[b47] Henkelman G. & Jónsson H. Improved tangent estimate in the nudged elastic band method for finding minimum energy paths and saddle points. J. Chem. Phys. 113, 9978–9985 (2000).

[b48] Henkelman G., Uberuaga B. P. & Jónsson H. A climbing image nudged elastic band method for finding saddle points and minimum energy paths. J. Chem. Phys. 113, 9901–9904 (2000).

[b49] Nakano A. A space–time-ensemble parallel nudged elastic band algorithm for molecular kinetics simulation. Comp. Phys. Commun 178, 280–289 (2008).

[b50] Santos, dos R. P. B., Perim E., Autreto P. A. S., Brunetto G. & Galvão D. S. On the unzipping of multiwalled carbon nanotubes. Nanotechnology 23, 465702 (2012). 23093108 10.1088/0957-4484/23/46/465702

[b51] Perim E., Autreto P. A. S., Paupitz R. & Galvão D. S. Dynamical aspects of the unzipping of multiwalled boron nitride nanotubes. Phys. Chem. Chem. Phys. 15, 19147 (2013). 23999943 10.1039/c3cp52701h

[b52] Plimpton S. Fast parallel algorithms for short-range molecular dynamics. J. Comput. Phys. 117, 1–19 (1995).

[b53] Jund P. & Jullien R. Molecular-dynamics calculation of the thermal conductivity of vitreous silica. Phys. Rev. B 59, 13707–13711 (1999).

[b54] Chantrenne P. & Barrat J.-L. Finite size effects in determination of thermal conductivities: comparing molecular dynamics results with simple models. J. Heat Transfer 126, 577–585 (2004).

