# Supplementary material for: Ambient solid-state mechano-chemical reactions between functionalized carbon nanotubes
Source: Nat Commun. 2015 Jun 15;6:7291. doi: 10.1038/ncomms8291 (PMC4490571; doi:10.1038/ncomms8291)
Supplement: Supplementary Figures, Supplementary Tables, Supplementary Note, Supplementary Methods and Supplementary References. — Supplementary Figures 1-5, Supplementary Tables 1-2, Supplementary Note 1, Supplementary Methods and Supplementary References [file ncomms8291-s1.pdf]

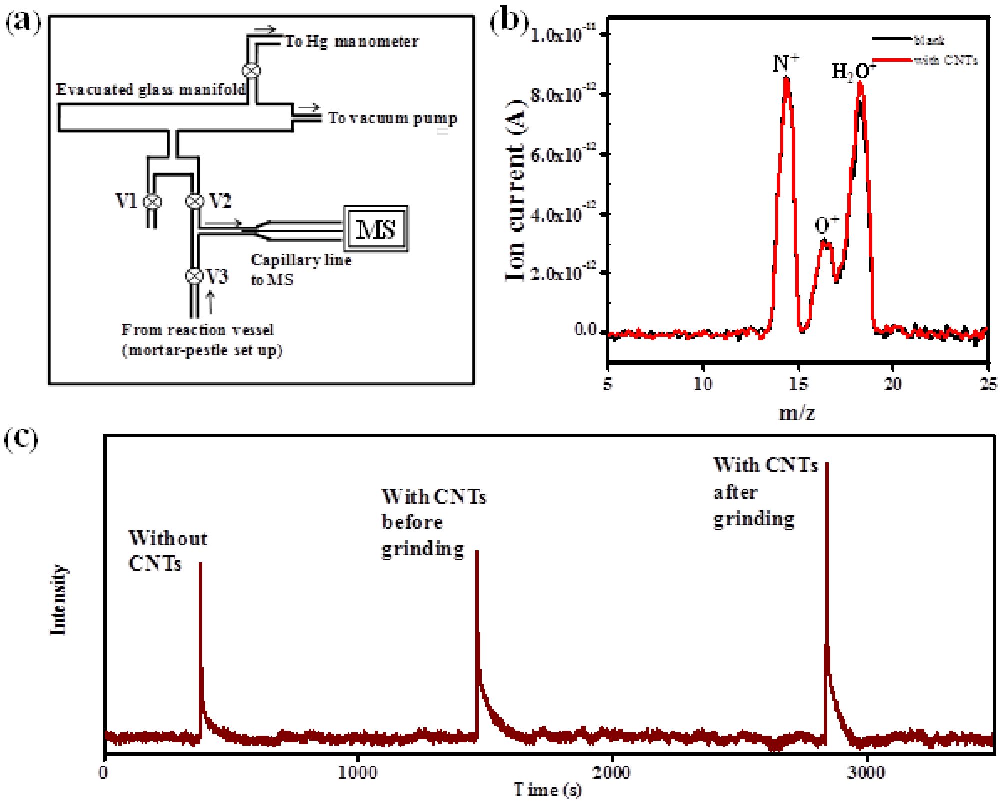

Supplementary Figure 1: (a) Schematic of the experimental set up for the online mass spectrometric detection of water during the solid state condensation reaction between MWCNTs, **(b)**Mass spectra showing the changes in N^+, O^+ and H_2O^+ intensities for the blank and the sample after grinding and **(c)** Ion current vs. time plots for H_2O^+ for blank (wihout MWCNTs), with MWCNTs before grinding and after grinding.

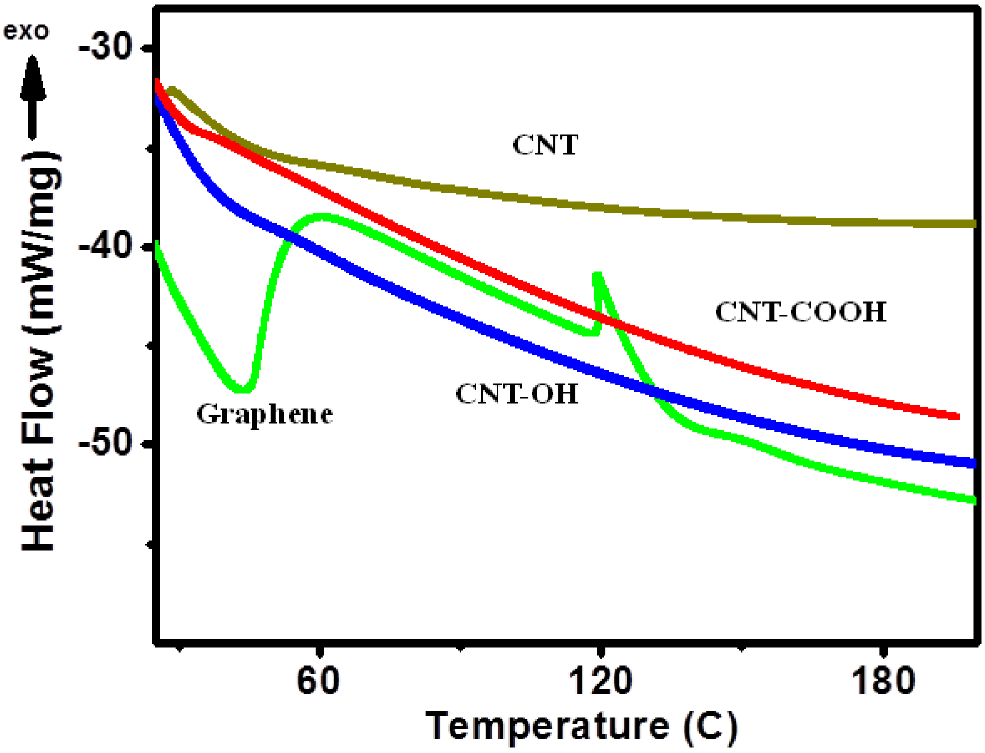

Supplementary Figure 2: DTA of individual unmixed CNTs and that of the graphene product**.** First peak is due to desorption of CO_2 while the second is due to desorption of water

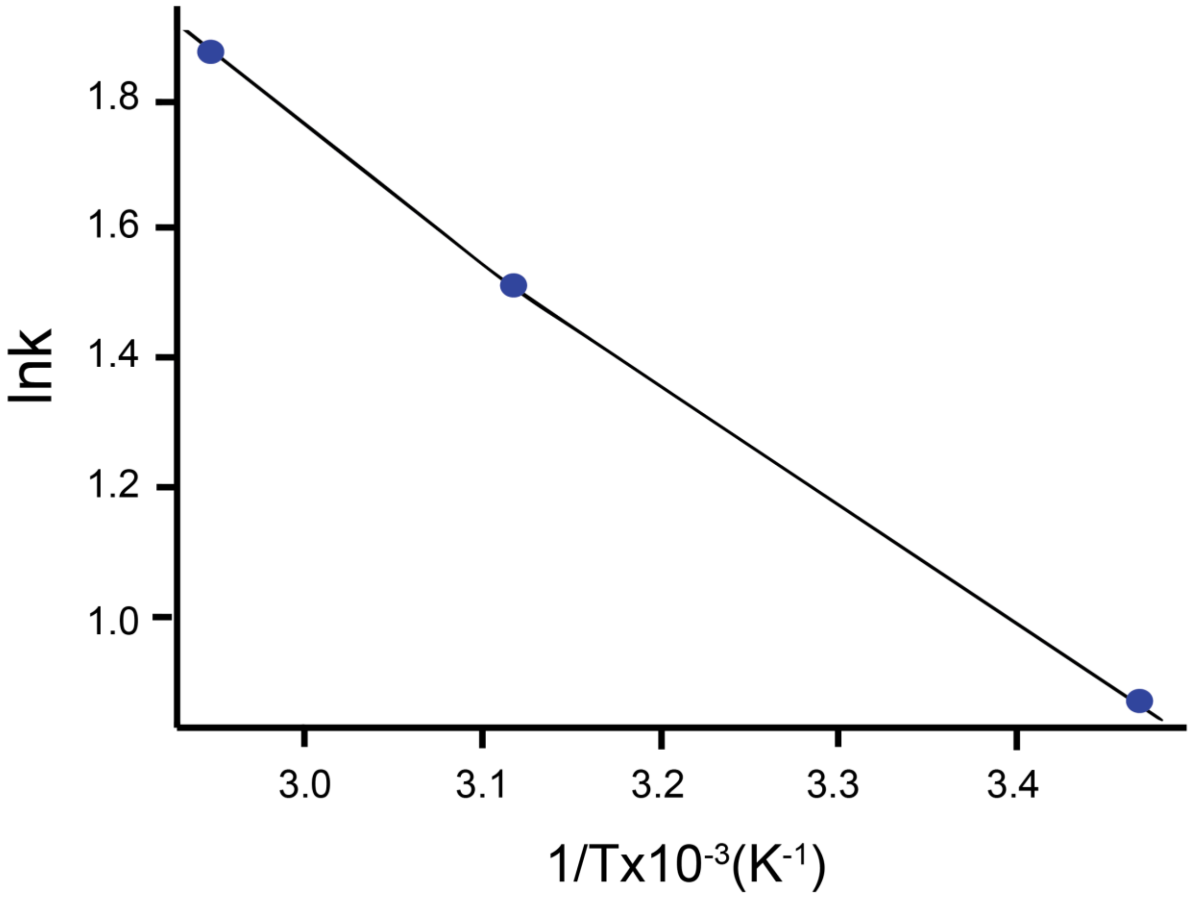

Supplementary Figure 3: In order to evaluate activation energy of the chemical reaction, a variable temperature Raman study was carried out. The two reactants, MWCNT-COOH and MWCNT-OH were mixed and ground at two temperatures (60, 70 ^{o}C) on hot plate. The reaction mixtures were quenched to room temperature before the Raman spectra were recorded; this was done for each temperature separately. The intensities of 2D Raman peaks have been used to calculate the rate constants as listed in Table.2.Arrhenius plot of lnk vs. 1/T.Slope =$-R/E_{act}=-2.00x10^3$

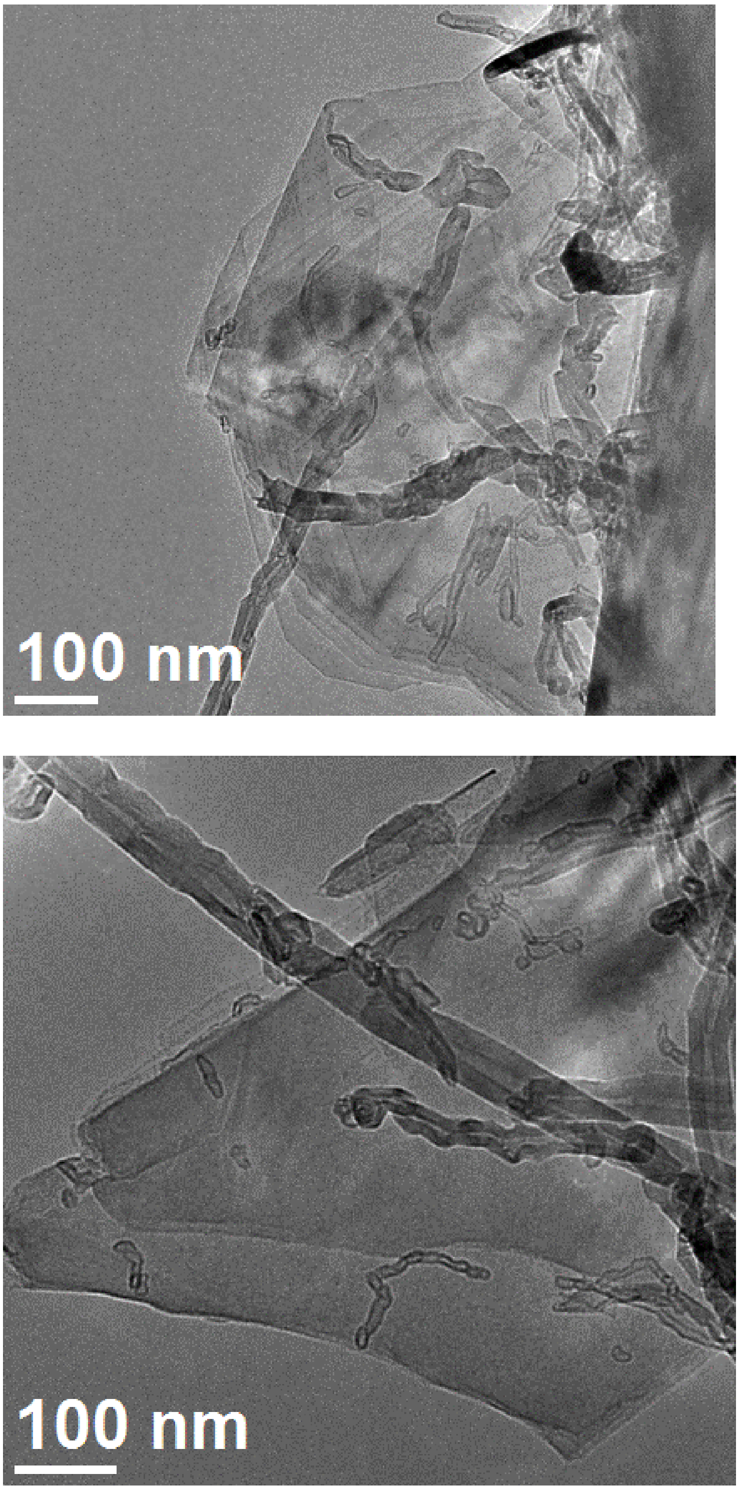

Supplementary Figure 4:. Bright field TEM images of graphene observed at different places and in different samples.

(a) d_{tubes}=3.5 Å

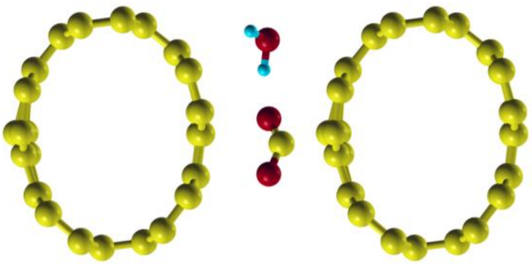

(b) d_{tubes}=4.5 Å

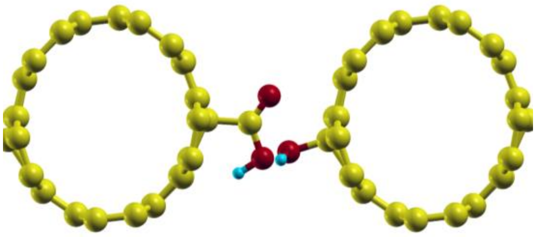

Supplementary Figure 5: (a) Geometric optimized structures for nanotubes initially placed at 3.5 Å, which results in the formation of H_2O and CO_2. (b) When the distance between the tubes is increased to 4.5 Å no reaction is observed and the functional groups remain attached to their parent tubes.

Supplementary Table 1: Table 1: % of Oxygen XPS data

Material	% Oxygen
MWCNT-OH	0.430
MWCNT-COOH	1.000
Unreactive mixture	0.715
Solid product after heating to a constant weight at 110 ^{o}C	0.280

Calculation of oxygen content:

Expected % oxygen after 60 % yield reaction =0.715 x 0.4=0.286

Amount of reacted oxygen due to simple esterification reaction =0.5/2=0.25%≪0.715-0.280 = 0.435% =observed amount of reacted oxygen. This represents ∼0.6x0.715=0.429% or expected 60% yield oxygen reacted.

Supplementary Table 2: Variable temperature 2D intensity data

**T, K**	**Intensity of the 2D Raman band (au)**	**ln k**	**1/T, K^{-1}**
286	2.3	0.833	3.497x10^{-3}
319	4.5	1.504	3.134x10^{-3}
341	7.0	1.946	2.932x10^{-3}

Supplementary Note 1

Details of the mass spectrometric measurements for the detection of water

For online monitoring of the reaction, we used a set up schematically shown in Supplementary Fig. 1a. Initially, a mortar and pestle were kept enclosed in a nitrile glove with the pestle protruding through one of its fingers. The glove was then connected to the mass spectrometer inlet using a glass tube through another finger of the glove. In order to ensure that this set up was adequate to detect tiny amounts of water vapor, we placed a drop of water in the mortar and allowed its vapors into the mass spectrometer. We could detect the expected features. To further confirm the adaptability of the method, we heated $CuSO_4.5H_2O$ at 63 ^oC (at which the loss of two water molecules happens). Here also, we were able to detect water lost from these crystals. These experiments showed that our method was adequate for monitoring the MWCNT reactions. For monitoring the reactions, the set-up was evacuated after keeping the reagents inside the reaction vessel and Valve 3 (see Supplementary Fig. 1a) was closed. After grinding the ingredients for 20 minutes to complete the reaction, Valve 3 was opened and the vapor phase in the reaction vessel was sampled. Changes in the partial pressures of the constituents were evaluated. A blank experiment was performed under the same conditions without the sample, but with equal time for evacuation and reaction. This was done to ensure that artifacts due to potential leaks were avoided. From a quantitative evaluation of the changes in partial pressures, the yield was calculated.

In main figure Fig. 2d, the first peak (for H_2O^+)/first steps (for N^+ and O^+) correspond to blank measurement while second peak/steps correspond to measurement after grinding MWCNTs. For measuring the blank, at first, we kept the valves 1 and 3 closed and 2 open, the whole sample line was then evacuated for 2 h through a glass manifold using a rotary pump (see Supplementary Fig. 1a). Then valve 2 was closed and allowed the residual gases

(N_2 and O_2) and water content present in the volume of this enclosure to mass spectrometer by opening valve 3. Since the gases and moisture enclosed in the reaction vessel enter the mass spectrometer chamber, there was an increase in N^+, O^+ and H_2O^+ intensities (see the first rise in intensity for all traces in the main figure Fig. 2d). After certain time interval, valve 3 was closed and sample line was evacuated for some time (by opening valve 2). Due to this, intensity of all ions drops to initial value (see the fall in intensity of all traces in the first steps/peak of the main figure Fig. 2d). The sample line was pumped for some more time (to reset the base intensities). This finishes the blank measurement. Then, valve 2 was closed; MWCNTs were put into the mortar, ground vigorously. Valve 2 was now closed, and valve 3 was opened allowing the gaseous products into the mass spectrometer capillary for measurement. This result in second rise in intensities of N^+, O^+ and H_2O^+ (see the second rise in intensities for all traces in the main figure Fig. 2d). Please note that intensity of second H_2O^+ peak (i.e. after grinding MWCNTs) is significantly higher compared to that of blank. This increase is due to the excess amount of water formed due to MWCNTs reaction. After some time, valve 3 was closed; sample line was again pumped, resulting in drop in intensities. This completes a typical experiment. Always, each experiment was carried out by doing a blank one first, followed by the actual sample measurement. Both these were done in a single run, of about one hour duration, to avoid errors that may result from variations in vacuum and the detector response. Mass spectrometer was baked at 100 ^oC for 24 h prior to any measurement and kept at baking condition during measurements. Also, to avoid condensation of moisture in the mass spectrometer capillary line, it was kept at 150 ^oC throughout the experiment.

We carried out Density Functional Theory (DFT)[1-3] geometry optimizations for model systems in order to investigate the reactions between the functional CNT groups and to validate the results obtained using ReaxFF. For these DFT calculations we did not carried out

a systematic study varying the tube distances. Due to the high computational cost, we just investigated two limit cases: (a) tubes closer enough (~3.5 Å) to be reactive, and (b) tubes well separated (~4.5Å), where no chemical reactions are expected to occur and (See Fig. S5). Our results showed that, as expected, the functional groups remain attached to the CNT when the distance is 4.5 Å (Fig. S5(b)). For the case of an initial 3.5 Å distance, the geometrical optimization processes lead the functional groups to react, yielding water and carbon dioxide (Fig. S5(a)). The obtained energy difference between the reactants and products was of 24.8 kcal/mol (indicating an exothermic reaction), this energy can be released as heat into the system.

Supplementary Methods

The DFT calculations were carried out within a local density approximation (LDA)[4] and using the Perdew-Wang functional[5] , as implemented in the Quantum Espresso code[6]. The wave functions were expanded using a standard Plane Wave (PW) basis set[7]. Wavefunctions up to 40 Ry of kinetic energy were considered. A grid of 6×6×1 in the k-space was sampled using the Monkhorst-Pack method[8].

Supplementary References

1. Fiolhais, C., Nogueira, F. & Marques, M. A. *A primer in density functional theory*. **620,** (Springer, 2003).
2. Koch, W. & Holthausen, M. C. *A chemist's guide to density functional theory*. (Wiley-VCH Verlag GmbH, 2000).
3. Martin, R. M. *Electronic Structure*. (Cambridge University Press, 2008).
4. Kohn, W. & Sham, L. J. Self-Consistent Equations Including Exchange and Correlation Effects. *Phys. Rev.***140,** A1133–A1138 (1965).
5. Perdew, J. P. & Wang, Y. Accurate and Simple Analytic Representation of the Electron-Gas Correlation-Energy. *Phys. Rev. B***45,** 13244–13249 (1992).
6. Giannozzi, P. *et al.* QUANTUM ESPRESSO: a modular and open-source software project for quantum simulations of materials. *J. Phys.: Condens. Matter***21,** 395502 (2009).
7. Dabo, I., Kozinsky, B., Singh-Miller, N. & Marzari, N. Electrostatics in periodic boundary conditions and real-space corrections. *Phys. Rev. B***77,** 115139 (2008).
8. Monkhorst, H. J. & Pack, J. D. Special Points for Brillouin-Zone Integrations. *Phys. Rev. B***13,** 5188–5192 (1976).
